# A population-based nomogram to individualize treatment modality for pancreatic cancer patients underlying surgery

**DOI:** 10.1038/s41598-023-31292-6

**Published:** 2023-03-24

**Authors:** Xiao-Ya Shi, Yan Wang, Xuan Zhou, Meng-Li Xie, Qian Ma, Gan-Xin Wang, Jing Zhan, Yi-Ming Shao, Bai Wei

**Affiliations:** 1 Department of Oncology, Liyuan Hospital, Tongji Medical College, Huazhong University of Science and Technology, 39 Yanhu Avenue, Wuchang District, Wuhan, 430077 Hubei Province China; 2 Department of Oncology, Tianyou Hospital Affiliated to Wuhan University of Science and Technology, Wuhan, Hubei Province China; 3 Department of Clinical Medicine, Jining Medical University, Jining, Shandong Province China

**Keywords:** Cancer, Medical research, Oncology

## Abstract

As the most aggressive tumor, TNM staging does not accurately identify patients with pancreatic cancer who are sensitive to therapy. This study aimed to identify associated risk factors and develop a nomogram to predict survival in pancreatic cancer surgery patients and to select the most appropriate comprehensive treatment regimen. First, the survival difference between radiotherapy and no radiotherapy was calculated based on propensity score matching (PSM). Cox regression was conducted to select the predictors of overall survival (OS). The model was constructed using seven variables: histologic type, grade, T stage, N stage, stage, chemotherapy and radiotherapy. All patients were classified into high- or low-risk groups based on the nomogram. The nomogram model for OS was established and showed good calibration and acceptable discrimination (C-index 0.721). Receiver operating characteristic curve (ROC) and DCA curves showed that nomograms had better predictive performance than TNM stage. Patients were divided into low-risk and high-risk groups according to nomogram scores. Radiotherapy is recommended for high-risk patients but not for low-risk patients. We have established a well-performing nomogram to effectively predict the prognosis of pancreatic cancer patients underlying surgery. The web version of the nomogram https://rockeric.shinyapps.io/DynNomapp/ may contribute to treatment optimization in clinical practice.

## Introduction

As one of the most aggressive and highly metastatic malignancies, pancreatic cancer ranks seventh among cancer-related deaths^1^. Surgical resection with adjuvant/neoadjuvant therapy offers hope for long-term survival or cure in patients with non-metastatic pancreatic cancer^2^. Local recurrence and metastasis of pancreatic cancer have brought new challenges to the treatment^3^. However, the most effective treatment mode is uncertain, and an effective predictive model is needed to evaluate patient prognosis accurately.

TNM staging focuses on the pathological tumor characteristics of patients eligible for surgery^4^. However, even among patients with the same disease stage, the prognosis can vary widely^5^. This population-based cancer staging method has great generality, but lacks guidance for different treatment modalities. The nomogram is a predictive model that uses a specific scoring system to assess the probability of survival of patients ^6^. Based on various combinations of serological markers, clinicopathological parameters, imaging features, genomic features or biomarkers, many predictive models have been established to predict the prognosis of pancreatic cancer at different stages^7–9^. Nevertheless, the data for the predictive model did not include patient treatment information. Therefore, there is an urgent need to develop an effective predictive model that integrates clinicopathology with therapy, especially in pancreatic cancer patients underlying surgery. In this study, post-match cohorts were created after propensity score matching (PSM)^10^.

Currently, gemcitabine-based combination therapy has improved survival in adjuvant therapy and in metastatic settings^11^. The combination of multiple treatment options, such as FOLFIRINOX treatment and radiotherapy, brings new hope to patients. We discuss the impact of multidisciplinary treatment on patient survival and identify characteristics of patients who benefit from radiation therapy. In addition, a nomogram was constructed from the pre-competition cohort to segregate the population into different risk groups by predictors and then estimated the effect of radiotherapy on the upper group. Based on the evaluation of the patients’ prognosis, the most appropriate individualized treatment plan is provided for the patient through this nomogram.

## Methods

### Patient population

Patient demographics, treatment information and tumor characteristics were extracted from SEER*Stat version 8.3.9. The inclusion criteria were as follows: (1) Patients with pancreatic cancer as the first and only cancer diagnosis. (2) The primary site of the tumor is limited to the International Classification of Diseases for Oncology, 3rd Edition (ICD-O-3) C25.0–C25.3 site codes, and histological codes are 8050–8089, 8500–8549, and 8140–8389 Oncological Disease Classification. (3) Patients are undergoing radical pancreatic cancer resection. (4)Patients with codes of 10–90 in RX Summ – Surg Prim Site (1998 +) and Site-specific surgery (1973–1997, with varying details by year and site) were classified into the postoperative group. Exclusion criteria were as follows: (1) Patients with second primary pancreatic cancer. (2) Patients with missing or incomplete treatment information, TNM stage (AJCC 7th Edition clinical stage), or other characteristics. (3) Patients with 0 months of follow-up (perioperative death).

### Study variables

This study screened data from the SEER database on patients diagnosed with pancreatic cancer between 2010 and 2015, and selected patients who had undergone surgery. The variables we analyzed included age at diagnosis, sex, race, primary tumor site, tumor grade, radiotherapy, chemotherapy, surgery, marital status at diagnosis, insurance status, TNM stage (American Joint Commission on Cancer [AJCC] 7th version), pathological type, and survival information. The study endpoint was OS, the duration from diagnosis to death from any cause or last follow-up.

### Statistical analysis

Pearson’s chi-square analysis was used to evaluate different clinical characteristics between different treatment regimens. All statistical tests were two-sided, and a *P* value ​​ < 0.05 was considered statistically significant. PSM was performed using the nearest neighbor matching method with a caliper of 0.001. 1:1 PSM^12^ was designed to reduce the selection bias of baseline variables between groups, including age, sex, race, marital, insurance, tumor site, histological type, grade, T, N, M stage, and treatment pattern twelve variables. After PSM, univariate and multivariate competing risks regression models were used to evaluate statistically significant variables for survival outcomes, and hazard ratios (HR) and corresponding 95% confidence intervals (CI) were calculated.

The forest plot for OS Cox analyses shows the effect of different variables on survival outcomes and subgroup analysis results. In addition, Kaplan–Meier estimates were performed to show whether radiotherapy affects survival outcomes. Patients of 8026 were randomized 7:3 to a training cohort (n = 5618) and a validation cohort (n = 2408) using a random sampling function.

Risk factors associated with survival were identified based on multivariate competing risk regression analysis and creation of nomograms. For the discrimination and calibration of nomograms in the training and validation cohorts, the C-index and area under the receiver operating characteristic curve (AUC) were calculated, and a calibration curve was drawn using a bootstrapping method involving 1000 resamples. Calibrations were performed at 1, 2, 3, and 5 years to compare predicted versus observed survival in pancreatic cancer patients underlying surgery.

A standard curve was generated using the bootstrap method. The cohort was tested 1000 times for internal validation. For the calibration curve, the closer the curve is to the grey reference line, the closer the predicted value is to the actual situation. DCA^13^ is a method to assess the clinical utility of alternative models by quantifying the net utility of different threshold probabilities and applying them to a standard plot. Both references were patient protocol (representing higher clinical cost) and no protocol (representing no clinical benefit).

In addition, the nomogram’s accuracy and clinical benefit rate were compared with the AJCC 7th edition staging system. A nomogram corresponding estimated the patient’s total score to the risk of survival for pancreatic cancer, and all patients were divided into high- and low-risk groups by the median risk score. Then, the survival analysis of patients with radiotherapy in the above groups was calculated separately.

All statistical analyses were performed using R4.2.0 software (The R Project for Statistical Computing, http://www.r-project.org). Two-sided *P* values ​​were considered statistically significant if *P* < *0.05*.

### Ethics approval and consent to participate

Our institutional ethics review board approved this study.

## Results

### Characteristics of patients and disease

**Figure 1:**
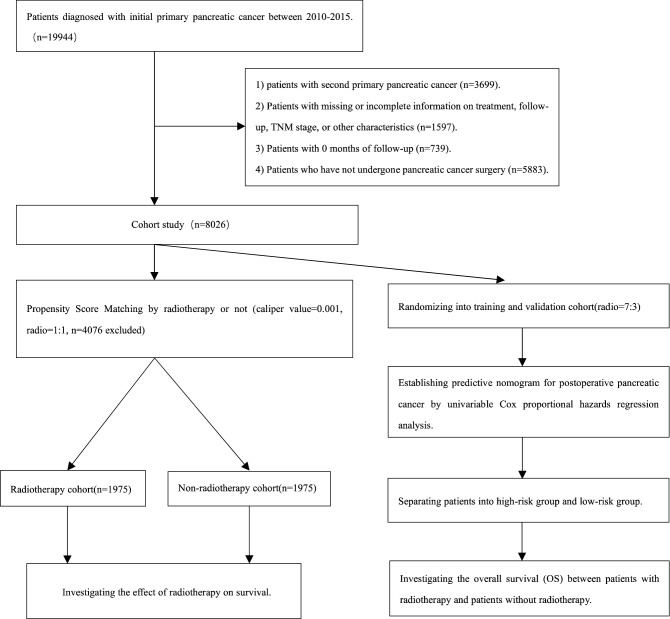
The data selection steps of the present study.

As Fig. 1 shows, a total of 8026 pancreatic cancer patients were identified from the SEER database. After performing PSM, a total of 4076 pts were eliminated at match conditions of ratio (1:1) and caliper value (0.001). A total of 3950 cases, including 1975 cases with radiotherapy and 1975 cases without radiotherapy, were finally chosen for further research. The patients’ characteristics before and after matching are shown in Table S1. After PSM, the distributions of all variables were similar and comparable. A similar distribution was also discovered in the no- radiotherapy group of the postmatch cohort.

### Survival analysis

Using the Kaplan–Meier survival curve, the OS of the nonradiotherapy group and the radiotherapy group before and after PSM was demonstrated by the log-rank test.

Especially after PSM, OS showed that patients with radiation therapy had a better prognosis than those without radiation. The median OS was 24 months (95% CI 0.460–0.506) and 26 months (95% CI 0.467–0.513) for the non-RT group and RT group, respectively (Figure S1A–B). In addition, survival analysis results showed that patients with Grade II, Grade III, N1, adenocarcinoma, ductal and lobular neoplasms, T3, and T4 benefited more from adjuvant radiotherapy (Figure S1C–F).

### Independent prognostic factors in the postmatch cohort

**Figure 2:**
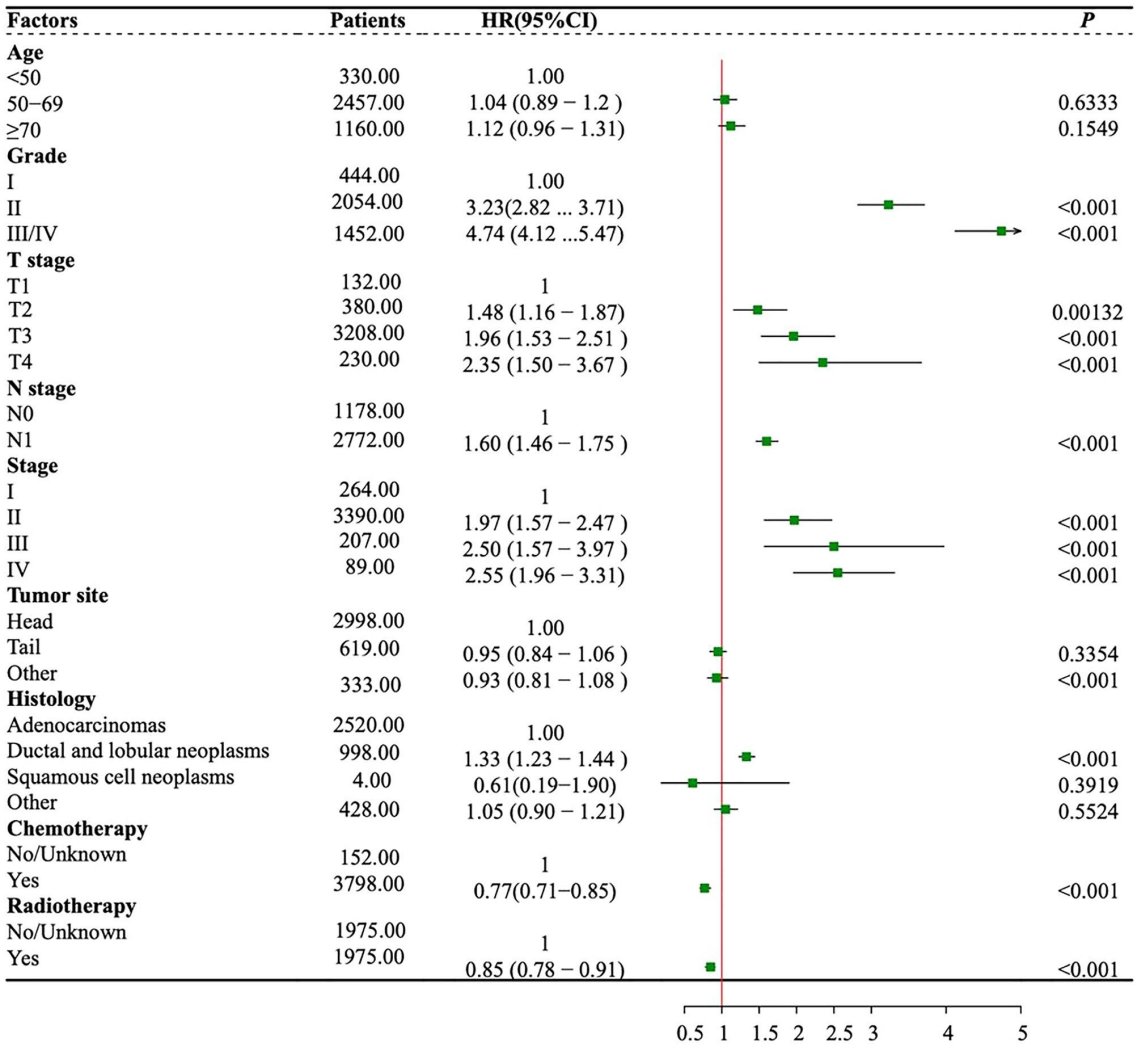
Hazard ratio in OS Cox analysis after PSM. *OS* Overall survival, *PSM* Propensity score matching, *HR* Hazard ratio, *CI* Confidence interval;

We performed univariate Cox proportional hazards regression analysis for fourteen potential factors and identified seven independent risk factors. Variables included T stage, N stage, tumor grade, histological subtype, stage, radiotherapy and chemotherapy. The multivariate Cox proportional hazards regression analysis results are clearly demonstrated in the forest plot in Fig. 2. In addition, we also made a forest plot to show the effect of radiotherapy in different subgroups of patients, but the results did not show that patients with the above characteristics were more likely to benefit from radiotherapy to improve their survival prognosis (Figure S2).

### Prognostic nomogram model establishment and validation in the prematch cohort

As Table S2 shows, in the training set, the univariate analysis showed that, marital status,age, tumor site, grade, TNM stage, stage, histological type, chemotherapy and radiotherapy were significantly correlated with pancreatic cancer (*P* < 0.05). Then, the multivariate Cox proportional hazards regression analysis indicated factors such as histological type, grade, T stage, N stage, stage, chemotherapy, and radiotherapy.

### Prognostic nomogram model establishment and validation

**Figure 3:**
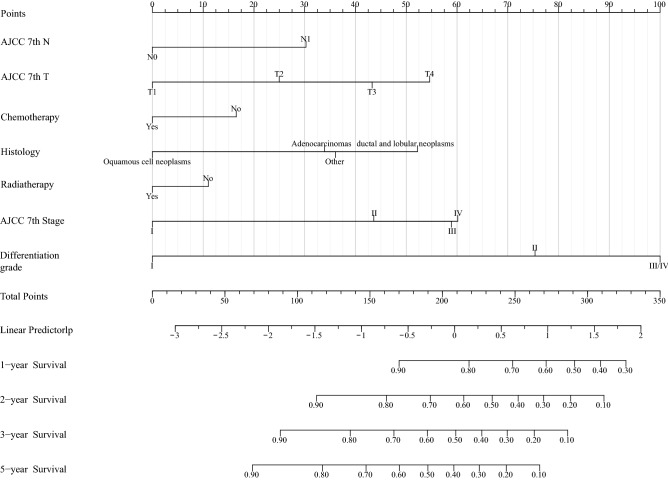
Nomogram to estimate the risk of prognosis for pancreatic cancer after surgery.

A nomogram was established based on independent predictors obtained by multivariate Cox proportional hazards regression analysis to display the 1‐year, 2‐year, 3‐year and 5‐year prognoses for pancreatic cancer after surgery (Fig. 3). The nomogram is provided through a free browser-based online calculator, and we have created a web version of the nomogram for ease of use, available at https://rockeric.shinyapps.io/DynNomapp/. We can predict patient survival prognostic risk by inputting the patient’s pathological variables (Figure S3).

**Figure 4:**
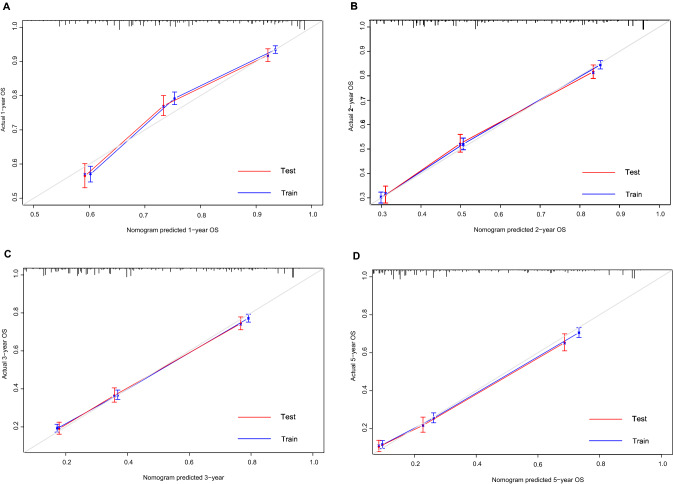
Calibration curves of the nomogram in the training cohort and validation cohorts. Calibration curves of 1-year (**A**), 2-year (**B**), 3-year (**C**) and 5-year (**D**) OS for patients in the training and validation cohorts. The plots along the 45° line indicate an appropriate calibration model, in which the predicted probabilities were identical to the actual outcomes. *OS* Overall survival.

**Figure 5:**
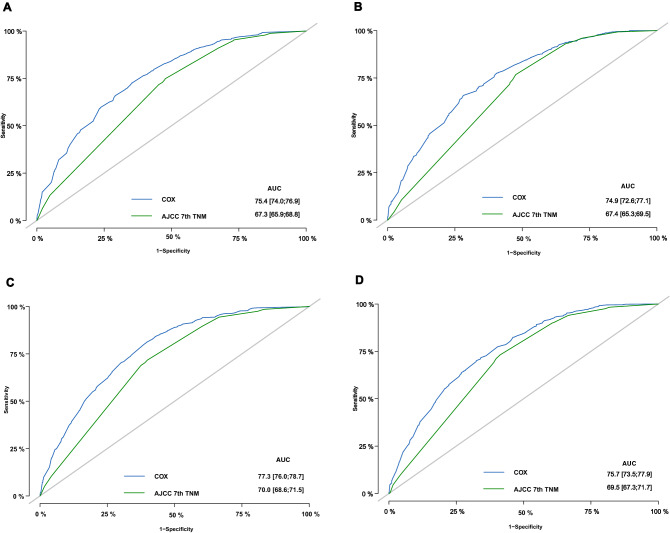

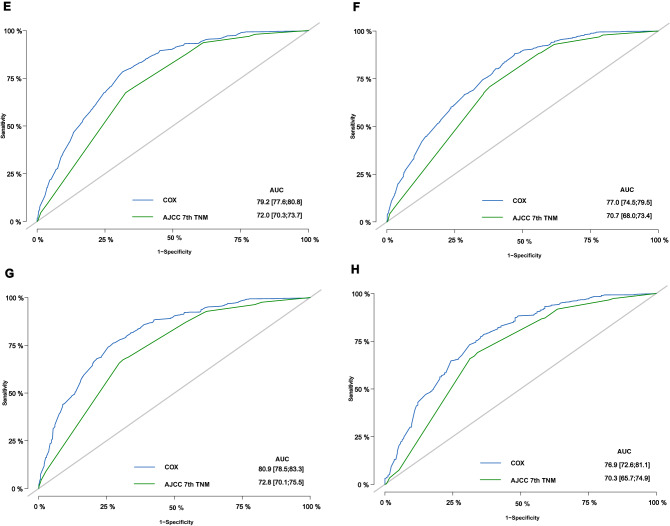
Receiver operating characteristic curve analysis in the training and validation cohorts of the nomograms and 7th edition AJCC-TNM staging system for predicting 1-, 2-, 3-, and 5-year OS. OS, overall survival.

**Figure 6:**
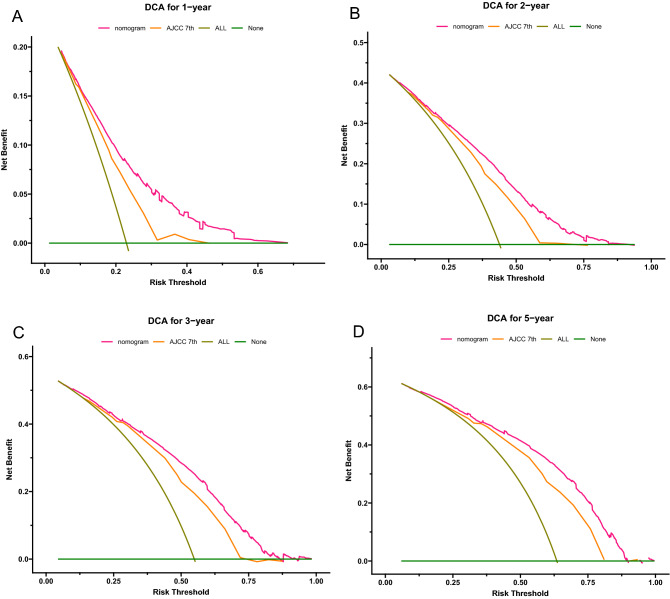
Decision curve analysis in the training cohort of the nomograms and 7th edition AJCC-TNM staging system for predicting 1-, 2-, 3-, and 5-year OS. *OS* Overall survival.

The bias-corrected C-index values in the training and validation cohorts were 0.721 (95% CI: 0.711–0.731) and 0.706 (95% CI: 0.690–0.722), respectively, indicating the moderate discrimination ability of the nomogram. Nomogram performance was quantified in terms of differentiation and calibration. The predictive accuracy of the nomogram was assessed by discrimination and calibration. The calibration curves of 1‐year, 2‐year, 3‐year, and 5‐year based on the training and validation cohorts are shown in Fig. 4(A–D). The calibration curves appeared to be very close to the ideal curve, representing good agreements between the nomogram‐predicted and the actual 1‐, 2‐, 3‐, and 5‐year prognoses for pancreatic cancer after surgery. The nomogram has good predictive accuracy and dependability in predicting pancreatic cancer after surgery. Similarly, the AUC of this study in the training cohorts and validation cohorts was also significantly higher than that of the TNM staging system (Fig. 5A–H). In the training cohort, DCA showed that the nomogram model is effective in clinical practice, with better clinical benefits than traditional TNM staging (Fig. 6A–D).

### Risk stratification and radiotherapy efficiency in different groups

**Figure 7:**
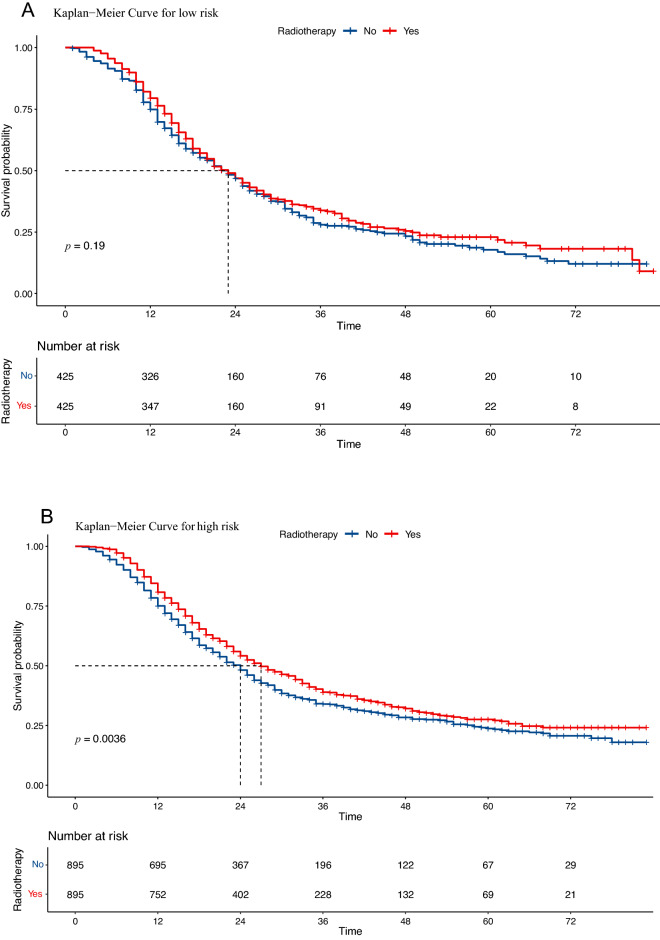
(**A**) Overall survival of pancreatic cancer patients with or without radiotherapy in the low‐risk group; (**B**) Overall survival of pancreatic cancer patients with or without radiotherapy in the high‐risk group.

The different variable risk scores were obtained from the nomogram, and all scores for each patient were summed to calculate the total score. The patients were dichotomized into high- and low-risk groups according to the risk score of the nomogram, and the results showed that the optimal cutoff value was 122.1 by X-tile software^14^ (Figure S4A–B). This study further compared whether patients in different risk groups could benefit from radiotherapy. The results showed no statistically significant difference in whether patients in the low-risk group benefited from radiotherapy (HR = 1.14; 95% CI: 0.97–1.35; p = 0.17; Fig. 7A), while patients in the high-risk groups benefited from radiotherapy (HR = 0.78; 95% CI: 0.71–0.85; *p *< 0.0001; Fig. 7B).

## Discussion

Due to the poor prognosis of pancreatic cancer, more than 90% of pancreatic cancer surgery patients will experience regional cancer recurrence, distant recurrence, or metastasis^15,16^.

Multidisciplinary therapy has been developed to include some combination of systemic chemotherapy, locoregional radiation, and surgery in selected patients with pancreatic cancer.

CRT with fluorouracil (FU) is considered the standard of care based on the Gastrointestinal Tumor Study Group (GITSG) trial and extensive case series analysis from Johns Hopkins and the Mayo Clinic^17,18^. However, conflicting results were obtained by the European Agency for Research and Cancer (EORTC)^19,20^ and the European Pancreatic Cancer Study (ESPAC-1)^21^. Both clinical trials showed a survival benefit with FU or gemcitabine, respectively, so chemotherapy alone as adjuvant therapy is advocated as standard in Europe. Currently, combination chemotherapy with FOLFIRINOX (leucovorin, 5-fluorouracil, irinotecan, and oxaliplatin) appears to be considered the most effective regimen for patients with pancreatic cancer. In addition, the MPACT study demonstrated improvement of overall survival in nearly one year by using nab-paclitaxel plus gemcitabine versus gemcitabine alone^22^. These studies have clinical and therapeutic implications. Radiotherapy plays an important role in controlling the local progression of tumors. With the advancement of modern precision radiotherapy technology, precision radiotherapy techniques such as intensity-modulated radiotherapy (IMRT), stereotactic body radiotherapy (SBRT) and proton beam therapy (PBT) have been implemented^23–25^. These novel treatment methods can effectively improve the dose distribution in the target area of ​​the radiotherapy plan, reduce the damage to normal tissues around the tumor, and achieve better local control of the tumor. The reasons why the local control benefit translates into a survival benefit may be multifactorial. The value of radiotherapy in addition to surgery for pancreatic cancer deserves further exploration. Insufficient evidence has not been found to determine the best combination of treatment options for individual patients. Therefore, there is a need to develop clinical prognostic models that can predict patient outcomes to potentially aid in developing comprehensive and patient-centered treatment regimens.

According to the study, we performed a PSM analysis to minimize the effect of between-group differences. After PSM, patients who received radiation therapy had better outcomes than those who did not. These data suggest that the survival benefit of RT can be complex, especially with modern RT techniques and various doses. For pancreatic cancer patients who require surgery, individualized treatment plans and accurate assessment of patient survival and prognosis are particularly important. Therefore, we developed a reliable system to accurately predict patient survival time, taking into account multiple prognostic factors.

We identified and modeled independent prognostic factors associated with survival by COX analysis. Our study shows that T stage, N stage, grade, stage and histological type are important risk factors. Radiation and chemotherapy are vital protective factors. Previous studies have attempted to model the prognosis of pancreatic cancer patients based on clinical features^26–28^. It has been evidenced that higher T and N stage, poor differentiation, stage and ductal adenocarcinoma are related to the worse prognosis. TNM staging evaluates patient prognosis by tumor size, lymph node metastasis, and distant metastasis^29^. However, disease progression is much more complex than the classical form. It is worth noting that, overall, the contribution of T or N staging to the survival of surgical patients mainly was no more significant than that of differentiation^30^.Our study also produced the same result. This means survival varies widely among pancreatic cancer surgery patients, even for the same TNM stage. Among other factors, the biological behavior of pancreatic cancer may be related to histological subtypes, particularly differences in survival rates for different histological types^31^. Ductal adenocarcinoma is a factor in poor survival in patients diagnosed with pancreatic cancer compared to other histological types^32^. The response of different histological exocrine pancreatic subtypes to tumor chemotherapy and radiotherapy remains unclear. In addition, clinical studies have shown that changes in serological indicators CA19-9, CEA, and CA125 are related to tumor progression and prognosis before and after surgery^33,34^. Vascular invasion, surgical margin status and lymph node positive rate are also important clinical indicators to predict the prognosis of patients^35,36^.Obviously, these need further research for clinical verification, so as to be better applied to clinical practice.

Unlike other studies, we not only developed and validated a prognostic nomogram, but further assessed the survival benefit of radiotherapy through risk stratification. In this study, the bias-corrected C-index values in the training and validation cohorts were 0.721 (95% CI: 0.711–0.731) and 0.706 (95% CI: 0.690–0.722), respectively, indicating good discriminative power of the nomogram. Baseline nomograms using available clinicopathological variables showed good performance in distinguishing patient outcomes at 1, 2, 3, and 5 years.

Based on the nomogram, a risk stratification system was formed, and all patients were clearly divided into two risk prognostic groups for further rational evaluation of comprehensive treatment. Furthermore, based on the nomogram, a risk stratification system was formed by which all patients were unambiguously divided into two risk prognostic groups. In addition, the model also screens patients for further rational comprehensive treatment through risk stratification. Some benefits of radiation therapy may be offset by side effects of diffuse radiation to nearby vital organs. This means that a subgroup should be defined to differentiate those who would benefit from radiotherapy. Through risk stratification analysis, we found that radiotherapy was beneficial to improve OS in high-risk group patients, but not in low-risk patients. Although the SEER database does not provide specific radiation information (irradiation extent and radiation dose), OS is sufficiently reliable and effective to assess the therapeutic effect of RT and could reflect the clinical effect of radiotherapy. In our risk stratification model constructed with a nomogram, the high-risk group was characterized by positive lymph nodes, moderately differentiated, poorly differentiated, adenocarcinoma, T3, and T4, and benefited from radiotherapy. The more aggressive cancer features of the high-risk group may result in residual tumor cells, but a better survival benefit with radiotherapy compared with the low-risk group. The reason may be that patients in the high-risk group achieved absolute local tumor control after radiotherapy, which translated to significant survival benefit. Therefore, for high-risk patients, radiotherapy techniques with low side effects should be used on the premise of ensuring the tolerance and effectiveness of treatment.

With this nomogram, physicians can evaluate the survival prognosis of pancreatic cancer patients and preliminarily select appropriate treatment options. In addition, we have developed an easy-to-use web calculator to make it easily accessible to the general public. This study provides a new perspective for individualized treatment of pancreatic cancer patients undergoing surgery. While acknowledging some limitations, the study has several strengths. The advantages of this study are: large population base, many variables included, PSM was used to adjust confounding factors. More importantly, the study described the population-level survival benefit of patients treated with radiotherapy by OS, and advanced a pair of models for stratified analysis, with good discrimination and internal validation.

Furthermore, in this retrospective study, unknown factors cannot be ruled out to have influenced the results. Therefore, more caution should be exercised when using predictive models to decide patient treatment options. With the emergence of new chemotherapy regimens and radiotherapy techniques, more factors will be included to improve the model, and prospective trials are needed to verify it.

Finally, there are limitations to the study. First, although PSM addressed treatment selection bias, there is still some potential for bias in this retrospective cohort study. Second, our analysis shared the limitations of the SEER datasets. The datasets are limited by the quality and availability of the original data. The duration and regimens of chemotherapy used are not available from SEER. Radiation information, such as technology, dose and sequence, are not provided. Finally, this is a retrospective study. Third, considering the sample size, the number of covariates included in the COX regression model is large, and some of them show collinearity. The predictive model should be validated in a large, multicenter pancreatic cancer clinical trials.

## Conclusion

In summary, it has higher accuracy, good clinical utility, and more precise prognostic prediction than traditional staging systems. Our nomogram can be used to predict survival and to assess the survival benefit of radiation therapy in different patients. Radiation therapy should be evaluated in conjunction with existing systemic chemotherapy regimens to facilitate individualized treatment.

## Supplementary Information

Supplementary Figure S1.

Supplementary Figure S2.

Supplementary Figure S3.

Supplementary Figure S4.

Supplementary Table S1.

Supplementary Table S2.

## Data Availability

All data are available at SEER database (https://seer.cancer.gov/). under name “Incidence—SEER 18 Regs Custom Data (with additional treatment fields), Apr 2022 Sub (2010–2015 varying)”.

## Abbreviations

SEER: Surveillance, epidemiology, and end results
OS: Overall survival
CI: Confidence interval
HR: Hazard ratio
PSM: Propensity score matching
C-index: The concordance index
ROC: Receiver operating characteristic curve
AUC: The receiver operating characteristic curve
DCA: Determination curve analysis
AJCC: American joint committee on cancer
RT: Radiotherapy
