# Supplementary material for: A population-based nomogram to individualize treatment modality for pancreatic cancer patients underlying surgery
Source: Sci Rep. 2023 Mar 24;13:4856. doi: 10.1038/s41598-023-31292-6 (PMC10038997; doi:10.1038/s41598-023-31292-6)
Supplement: Supplementary file 1 — Supplementary Figure S1. [file 41598_2023_31292_MOESM1_ESM.docx]

A B


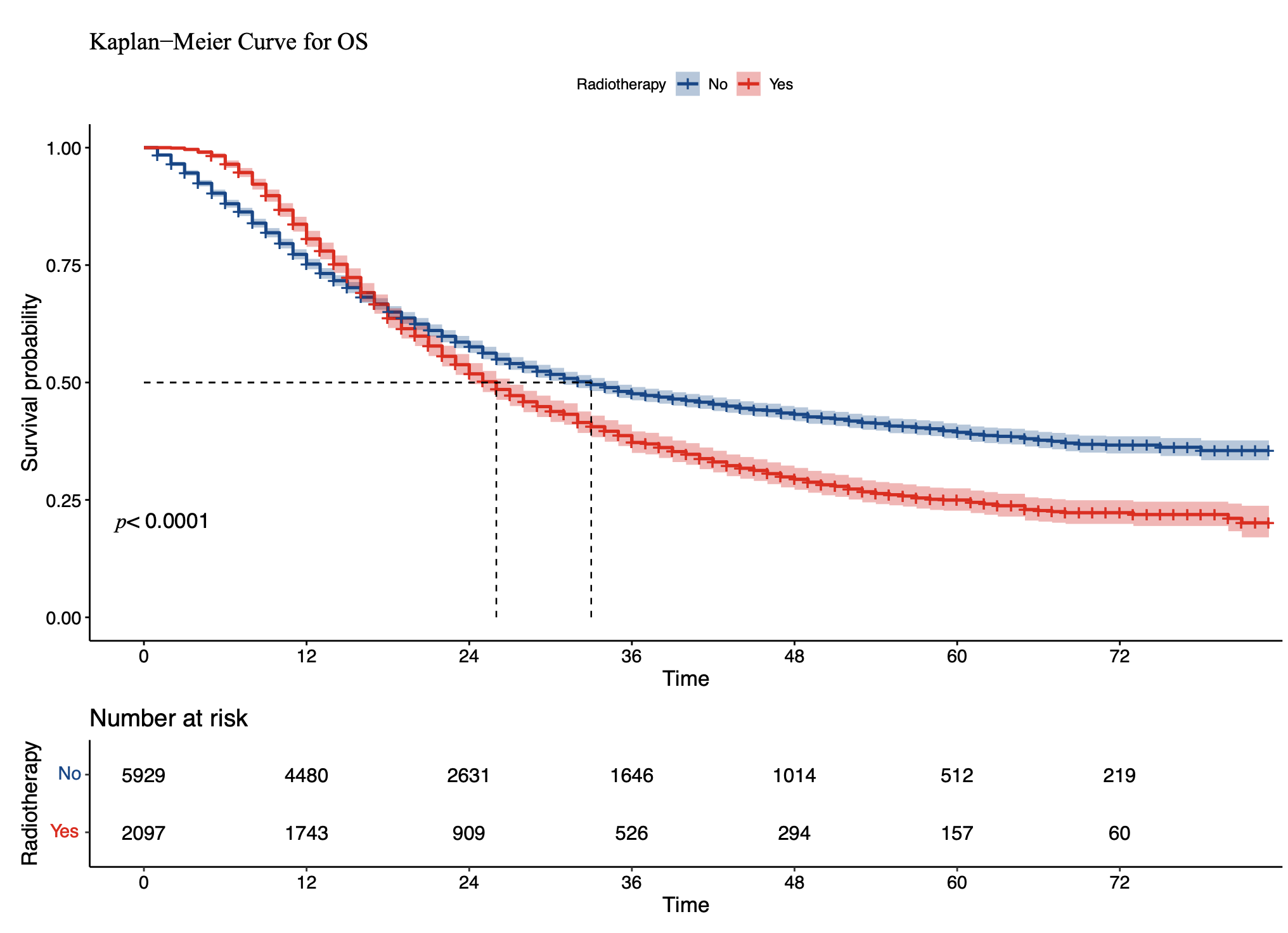

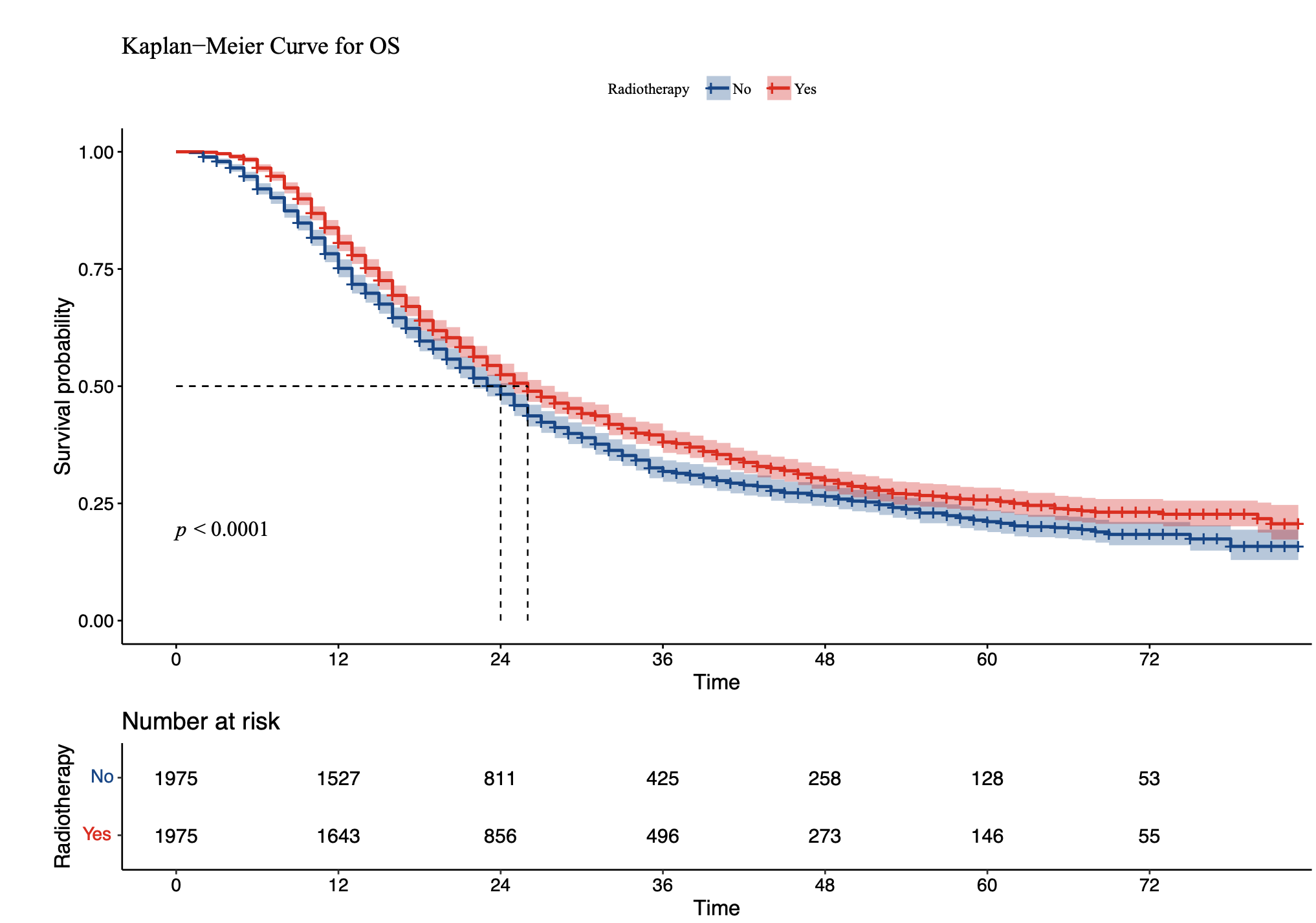


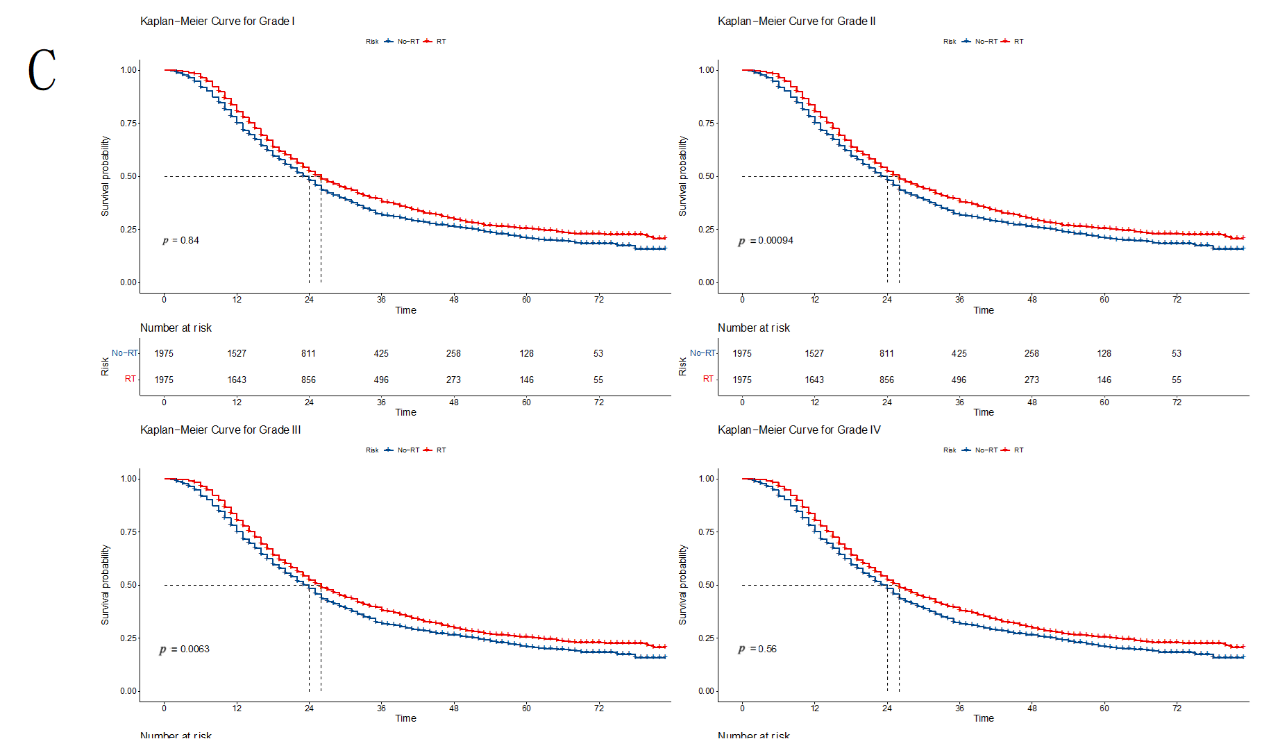


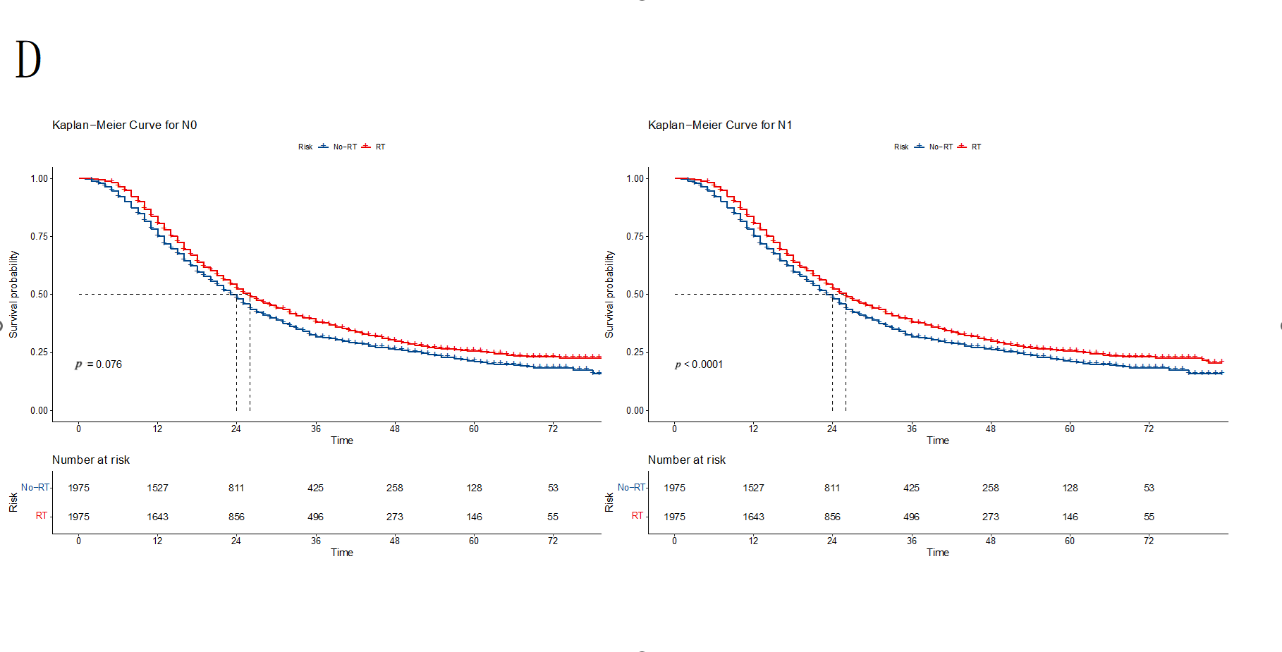


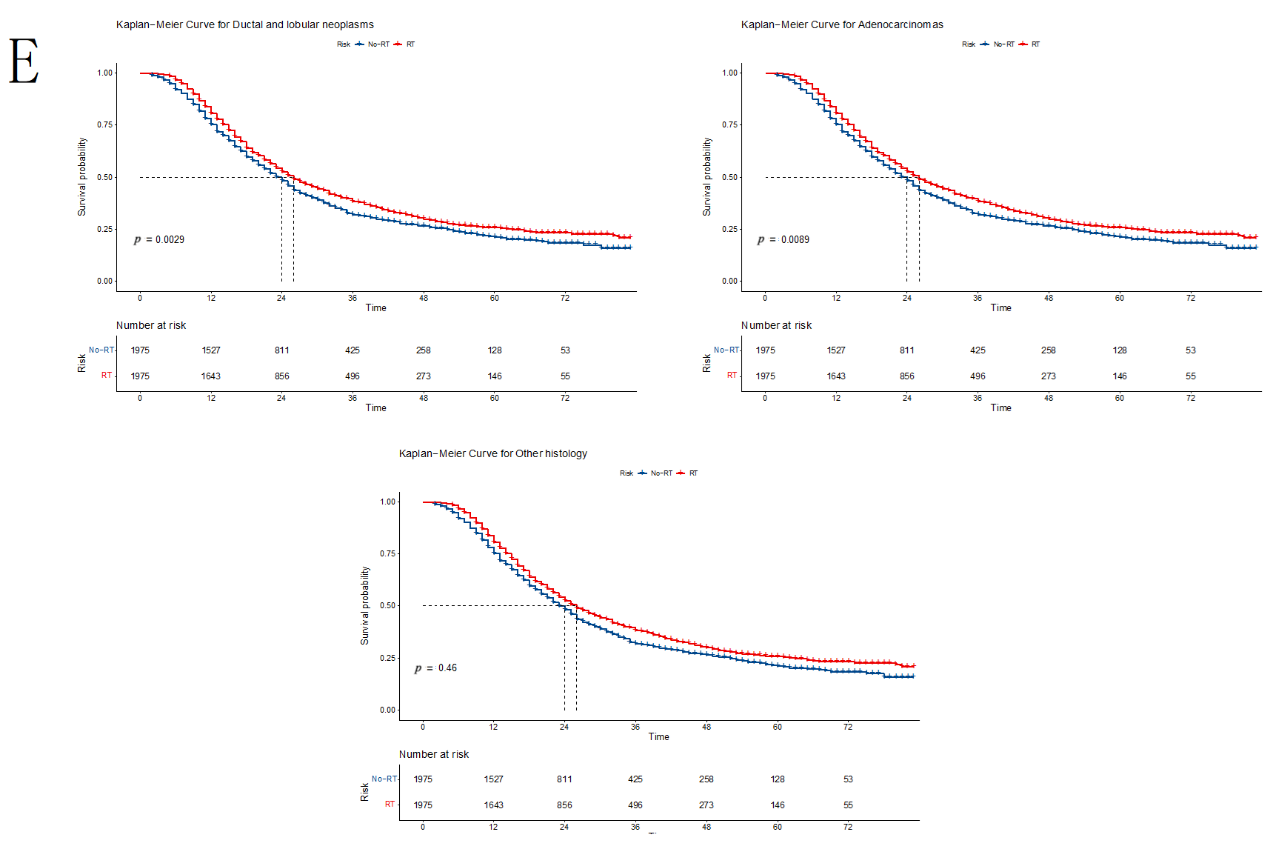


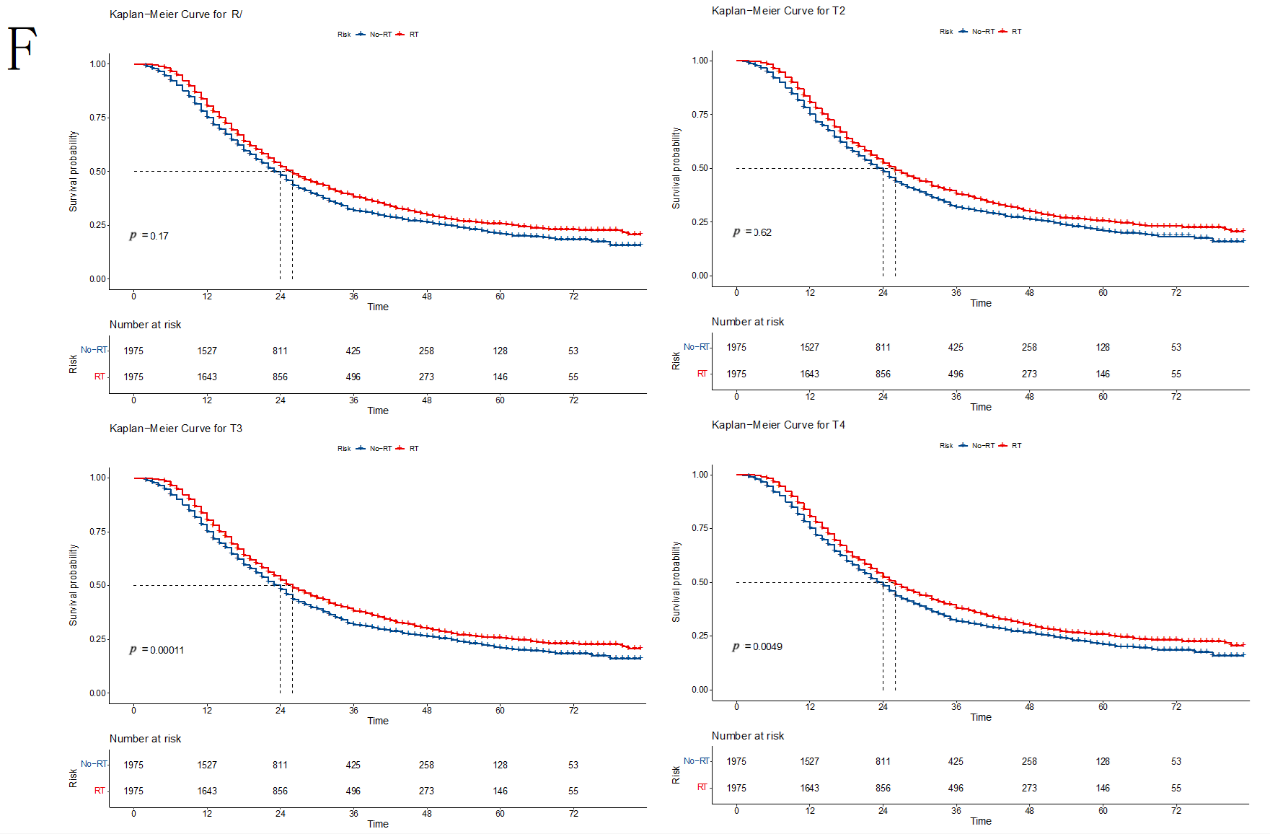


Figure S1 Kaplan–Meier survival analyses based on the non-RT group and RT group before and after PSM

A. Survival curves of the whole population in the non-RT group and RT group before PSM.

B. Survival curves of the whole population in the non-RT group and RT group after PSM.

C. Survival curves of patients with different grades in the non-RT group and RT group after PSM.

D. Survival curves of patients with different N stages in the non-RT group and RT group after PSM.

E. Survival curves of patients with different histological types in the non-RT group and RT group after PSM.

F. Survival curves of patients with different T stages in the non-RT group and RT group after PSM.

RT, radiotherapy; OS, overall survival.

Grade: Ⅰ, well differentiation; Ⅱ, moderate differentiation; Ⅲ, poor differentiation; Ⅳ, undifferentiation.
