# Supplementary material for: A population-based nomogram to individualize treatment modality for pancreatic cancer patients underlying surgery
Source: Sci Rep. 2023 Mar 24;13:4856. doi: 10.1038/s41598-023-31292-6 (PMC10038997; doi:10.1038/s41598-023-31292-6)
Supplement: Supplementary file 2 — Supplementary Figure S2. [file 41598_2023_31292_MOESM2_ESM.docx]

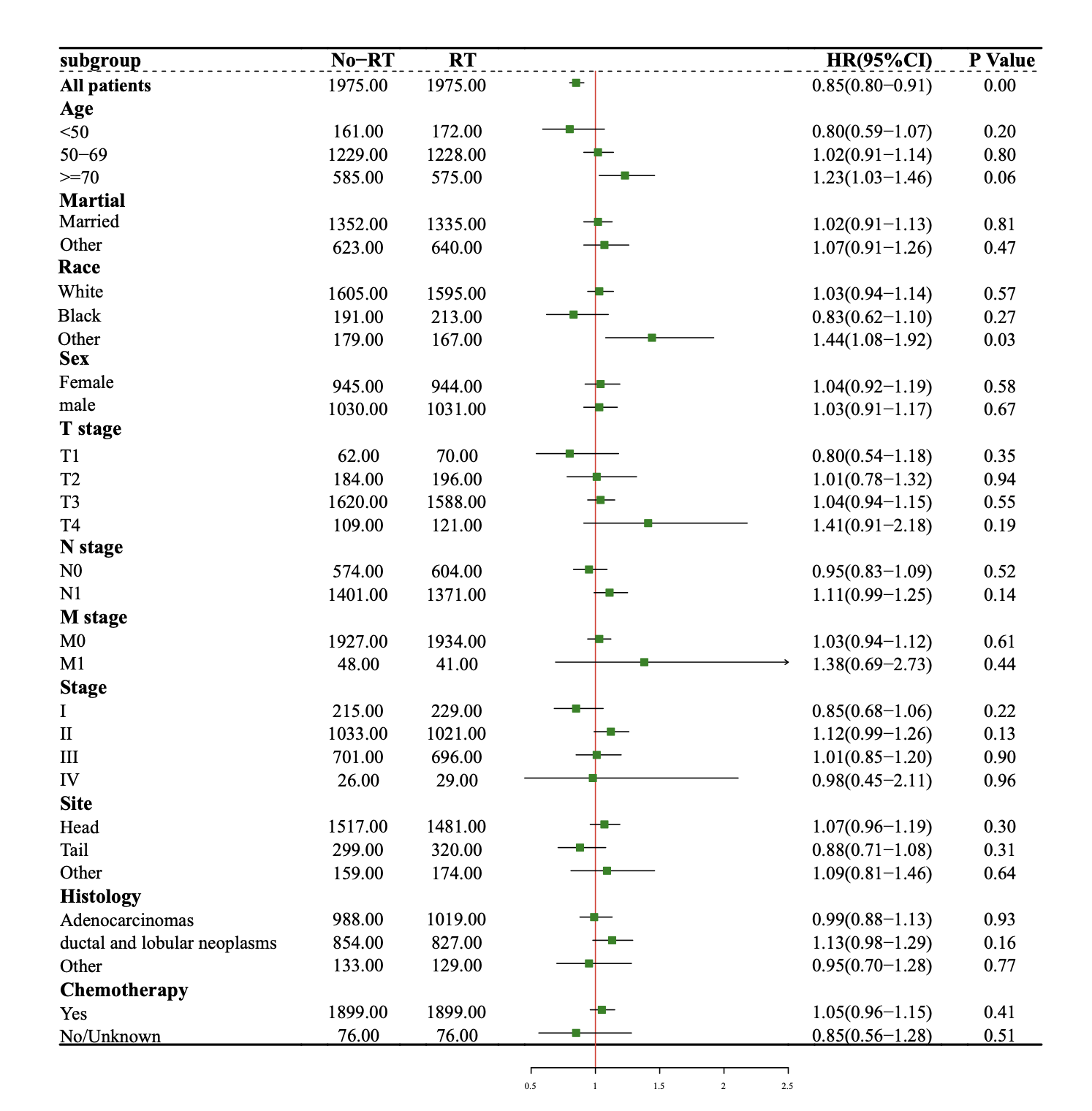


Figure S2 Hazard ratio forest plot of RT versus No-RT in each subgroup after PSM.

OS, overall survival; PSM, propensity score matching; HR, hazard ratio; CI, confidence interval; No-RT, No radiotherapy;

RT, Radiotherapy
