# Supplementary figures and images for: A population-based nomogram to individualize treatment modality for pancreatic cancer patients underlying surgery
Source: Sci Rep. 2023 Mar 24;13:4856. doi: 10.1038/s41598-023-31292-6 (PMC10038997; doi:10.1038/s41598-023-31292-6)

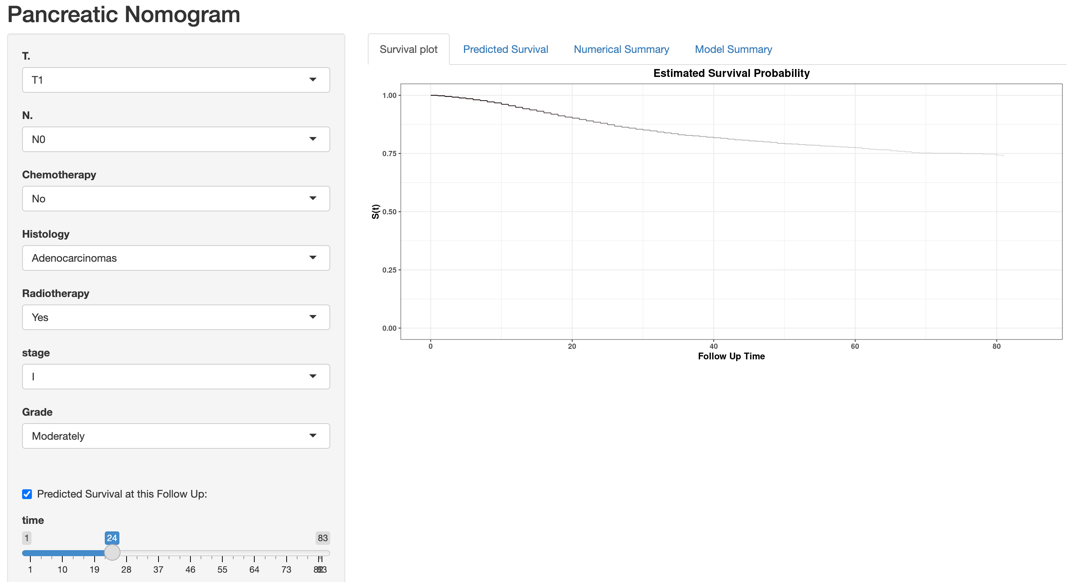


Figure S3 A web-based calculator predicts resectable OS in pancreatic cancer patients.

Supplement: Supplementary file 3 — Supplementary Figure S3. [file 41598_2023_31292_MOESM3_ESM.docx]
