# Supplementary material for: A population-based nomogram to individualize treatment modality for pancreatic cancer patients underlying surgery
Source: Sci Rep. 2023 Mar 24;13:4856. doi: 10.1038/s41598-023-31292-6 (PMC10038997; doi:10.1038/s41598-023-31292-6)
Supplement: Supplementary file 4 — Supplementary Figure S4. [file 41598_2023_31292_MOESM4_ESM.docx]

Figure S4 X-tile analysis for risk stratification: (A) The optimal cutoff value; (B) Numbers of patients in low- and high-risk subsets.
