# Supplementary material for: A population-based nomogram to individualize treatment modality for pancreatic cancer patients underlying surgery
Source: Sci Rep. 2023 Mar 24;13:4856. doi: 10.1038/s41598-023-31292-6 (PMC10038997; doi:10.1038/s41598-023-31292-6)
Supplement: Supplementary file 5 — Supplementary Table S1. [file 41598_2023_31292_MOESM5_ESM.docx]

Table S1 Clinical characteristics before and after propensity score matching

|  | **Before matching** | | | **After matching** | | |
| --- | --- | --- | --- | --- | --- | --- |
| **Variables** | **No radiation (N=5929)** | **Radiation**  **(N=2097)** | ***P* value** | **No radiation (N=5929)** | **Radiation**  **(N=2097)** | ***P* value** |
| **Age** |  |  |  |  |  |  |
| <50 | 706 (11.90%) | 187 (8.90%) | <0.001 | 161 (8.2%) | 172 (8.7%) | 0.798 |
| 50-69 | 3214 (54.20%) | 1324 (63.10%) |  | 1229 (62.2%) | 1228 (62.2%) |  |
| ≥70 | 2009 (33.90%) | 586 (27.90%) |  | 585 (29.6%) | 575 (29.1%) |  |
| **Race** |  |  |  |  |  |  |
| Black | 603(10.20%) | 229 (10.90%) | 0.052 | 191 (9.7%) | 213 (10.8%) | 0.441 |
| Other | 585 (9.90%) | 171 (8.20%) |  | 179 (9.1%) | 167 (8.5%) |  |
| White | 4741(80.00%) | 1697 (80.90%) |  | 1605 (81.3%) | 1595 (80.8%) |  |
| **Sex** |  |  |  |  |  |  |
| Female | 2975 (50.20%) | 991 (47.30%) | 0.022 | 945 (47.8%) | 944 (47.8%) | 1 |
| Male | 2954 (49.80%) | 1106 (52.70%) |  | 1030 (52.2%) | 1031 (52.2%) |  |
| **Marital** |  |  |  |  |  |  |
| Married | 3846 (64.90%) | 1429 (68.10%) | 0.007 | 1352 (68.5%) | 1335 (67.6%) | 0.585 |
| Other | 2083 (35.10%) | 668 (31.90%) |  | 623 (31.5%) | 640 (32.4%) |  |
| **Insurance** |  |  |  |  |  |  |
| Insured | 5727 (96.60%) | 2036 (97.10%) | 0.290 | 1922 (97.3%) | 1917 (97.1%) | 0.873 |
| Uninsured | 160 (2.70%) | 44 (2.10%) |  | 41 (2.1%) | 44 (2.2%) |  |
| unknown | 42 (0.70%) | 17 (0.80%) |  | 12 (0.6%) | 14 (0.7%) |  |
| **Site** |  |  |  |  |  |  |
| Head | 3469 (58.50%) | 1597 (76.20%) | <0.001 | 1517 (76.8%) | 1481 (75.0%) | 0.404 |
| Other | 672 (11.30%) | 178 (8.50%) |  | 159 (8.1%) | 174 (8.8%) |  |
| Tail | 1788 (30.20%) | 322 (15.40%) |  | 299 (15.1%) | 320 (16.2%) |  |
| **Histology** |  |  |  |  |  |  |
| Adenocarcinomas | 3594 (60.60%) | 1106 (52.70%) | <0.001 | 988 (50.0%) | 1019 (51.6%) | 0.616 |
| Ductal and lobular neoplasms | 1879 (31.70%) | 861 (41.10%) |  | 854 (43.2%) | 827 (41.9%) |  |
| Other | 449 (7.60%) | 137 (6.53%) |  | 140 (0.70%) | 129 (6.5%) |  |
| **Grade** |  |  |  |  |  |  |
| I | 1805 (30.40%) | 231 (11.00%) | <0.001 | 215 (10.9%) | 229 (11.6%) | 0.874 |
| Ⅱ | 2383 (40.20%) | 1114 (53.10%) |  | 1033 (52.3%) | 1021 (51.7%) |  |
| Ⅲ | 1637 (27.60%) | 723 (34.50%) |  | 701 (35.5%) | 696 (35.2%) |  |
| Ⅳ | 104 (1.80%) | 29 (1.40%) |  | 26 (1.3%) | 29 (1.5%) |  |
| **Stage** |  |  |  |  |  |  |
| I | 1497 (25.20%) | 138 (6.60%) | <0.001 | 126 (6.4%) | 138 (7.0%) | 0.573 |
| II | 3781 (63.80%) | 1783 (85.00%) |  | 1704 (86.3%) | 1686 (85.4%) |  |
| III | 171 (2.90%) | 135 (6.40%) |  | 97 (4.9%) | 110 (5.6%) |  |
| IV | 480 (8.10%) | 41 (2.00%) |  | 48 (2.4%) | 41 (2.1%) |  |
| **T stage** |  |  |  |  |  |  |
| T1 | 916 (15.40%) | 70 (3.30%) | <0.001 | 62 (3.1%) | 70 (3.5%) | 0.613 |
| T2 | 1011 (17.10%) | 197 (9.40%) |  | 184 (9.3%) | 196 (9.9%) |  |
| T3 | 3788 (63.90%) | 1684 (80.30%) |  | 1620 (82.0%) | 1588 (80.4%) |  |
| T4 | 214 (3.60%) | 146 (7.00%) |  | 109 (5.5%) | 121 (6.1%) |  |
| **N stage** |  |  |  |  |  |  |
| N0 | 2778 (46.90%) | 626 (29.90%) | <0.001 | 574 (29.1%) | 604 (30.6%) | 0.313 |
| N1 | 3151 (53.10%) | 1471 (70.10%) |  | 1401 (70.9%) | 1371 (69.4%) |  |
| **M stage** |  |  |  |  |  |  |
| M0 | 5449 (91.90%) | 2056 (98.00%) | <0.001 | 1927 (97.6%) | 1934 (97.9%) | 0.52 |
| M1 | 480 (8.10%) | 41 (2.00%) |  | 48 (2.4%) | 41 (2.1%) |  |
| **Chemotherapy** |  |  |  |  |  |  |
| No/Unknown | 3192 (53.8%) | 76 (3.6%) | <0.001 | 76 (3.8%) | 76 (3.8%) | 1 |
| Yes | 2737 (46.2%) | 2021 (96.4%) |  | 1899 (96.2%) | 1899 (96.2%) |  |

Grade: Ⅰ, well differentiation; Ⅱ, moderate differentiation; Ⅲ, poor differentiation; Ⅳ, undifferentiation.
