# Supplementary material for: A population-based nomogram to individualize treatment modality for pancreatic cancer patients underlying surgery
Source: Sci Rep. 2023 Mar 24;13:4856. doi: 10.1038/s41598-023-31292-6 (PMC10038997; doi:10.1038/s41598-023-31292-6)
Supplement: Supplementary file 6 — Supplementary Table S2. [file 41598_2023_31292_MOESM6_ESM.docx]

Table S2 Univariate and multivariate analysis of overall survival in patients with resectable pancreatic cancer.

|  | Univariate |  | Multivariate |  |
| --- | --- | --- | --- | --- |
| Characteristics | **HR^a^.CI95^b^** | ***P* value** | **HR^a^.CI95^b^** | ***P* value** |
| Age |  |  |  |  |
| <50 | 1 |  | 1 |  |
| 50-69 | 1.83 (1.62-2.07) | <0.001 | 1.3 (0.94 - 1.47) | <0.001 |
| ≥70 | 2.7 (2.38-3.06) | <0.001 | 1.67 (0.97 - 1.9) | <0.001 |
| Sex |  |  |  |  |
| Female | 1 |  |  |  |
| Male | 1.01 (0.95-1.08) | 0.677 |  |  |
| Insurance |  |  |  |  |
| Insured | 1 |  |  |  |
| Uninsured | 0.95 (0.78-1.16) | 0.638 |  |  |
| Unknown | 0.99 (0.69-1.43) | 0.971 |  |  |
| Marital |  |  |  |  |
| Married | 1 |  | 1 |  |
| Other | 1.12 (1.05-1.19) | <0.001 | 1.11 (0.95 - 1.19) | <0.001 |
| Race |  |  |  |  |
| Black | 1 |  |  |  |
| Other | 0.94 (0.82-1.09) | 0.421 |  |  |
| White | 1.09 (0.99-1.21) | 0.082 |  |  |
| Tumor Site |  |  |  |  |
| Head | 1 |  | 1 |  |
| Other | 0.61 (0.55-0.68) | <0.001 | 0.86 (0.78 – 1.06) | 0.0086 |
| Tail | 0.46 (0.42-0.5) | <0.001 | 0.77 (0.71 - 1.04) | <0.001 |
| Stage |  |  |  |  |
| Ⅰ | 1 |  | 1 |  |
| Ⅱ | 6.15 (5.4-7) | <0.001 | 1.72 (1.42 - 2.09) | <0.001 |
| Ⅲ | 8.66 (7.22-10.39) | <0.001 | 1.96 (1.31 - 2.94) | 0.0011 |
| Ⅳ | 6.87 (5.83-8.11) | <0.001 | 2.4 (1.93 - 2.99) | <0.001 |
| T stage |  |  |  |  |
| T1 | 1 |  | 1 |  |
| T2 | 2.52 (2.07-3.06) | <0.001 | 1.54 (1.26 - 1.89) | <0.001 |
| T3 | 6.97 (5.87-8.26) | <0.001 | 2.1 (1.7 - 2.59) | <0.001 |
| T4 | 9.29 (7.56-11.42) | <0.001 | 2.91 (1.97 - 4.31) | <0.001 |
| N stage |  |  |  |  |
| N0 | 1 |  | 1 |  |
| N1 | 2.72 (2.54-2.91) | <0.001 | 1.56 (1.44 - 1.68) | <0.001 |
| M stage |  |  |  |  |
| M0 | 1 |  |  |  |
| M1 | 1.45 (1.3-1.62) | <0.001 |  |  |
| Grade ^c^ |  |  |  |  |
| I | 1 |  | 1 |  |
| Ⅱ | 4.41 (3.95-4.92) | <0.001 | 3.23 (2.82 – 3.71) | <0.001 |
| Ⅲ/Ⅳ | 4.87 (6.15-7.68) | <0.001 | 4.74 (4.12 –5.47) | <0.001 |
| Histology |  |  |  |  |
| Adenocarcinomas | 1 |  | 1 |  |
| Ductal and lobular neoplasms | 1.85 (1.74-1.97) | <0.001 | 1.29 (1.21 - 1.38) | <0.001 |
| Other | 1.18 (1.04-1.33) | 0.01 | 1.05 (0.92 - 1.19) | 0.4632 |
| Radiotherapy |  |  |  |  |
| No | 1 |  | 1 |  |
| Yes | 1.22 (1.14-1.3) | <0.001 | 0.85 (0.79 - 0.91) | <0.001 |
| Chemotherapy |  |  |  |  |
| No | 1 |  | 1 |  |
| Yes | 1.81 (1.69-1.93) | <0.001 | 0.78 (0.72 - 0.84) | <0.001 |

HR, hazard ratio. CI, confidence interval.

Grade: Ⅰ, well differentiation; Ⅱ, moderate differentiation; Ⅲ, poor differentiation; Ⅳ, undifferentiation.
